# Cultures of birthing in transition

**DOI:** 10.1007/s11614-022-00476-1

**Published:** 2022-02-23

**Authors:** Sabine Flick, Franziska Marek, Friederike M. Hesse

**Affiliations:** 1 Hochschule Fulda, Gebäude 26, Raum 108, Leipziger Straße 123, 36037 Fulda, Germany; 2 Klinik für Psychiatrie und Psychotherapie II, Sektion Public Mental Health, Universität Ulm, Parkstraße 11, 89073 Ulm, Germany; 3 Fachbereich 4—Soziale Arbeit und Gesundheit, Frankfurt University of Applied Sciences, Nibelungenplatz 1, 60318 Frankfurt am Main, Germany

Being born and giving birth have received little sociological appreciation so far, although they are fundamental everyday events. In the last decade, the German language literature on the subject comprises only one extensive publication and a few articles (Hirschauer et al. 2014; Villa et al. 2011; Rose and Schmied-Knittel 2011; Rose 2010). It is astonishing that there are so few empirical and theoretical studies of birth and birthing. Theories involving the links between nature, body, and culture, which have been broadly examined in recent years in the context of Science and Technology Studies and New Materialism, offer versatile approaches to the phenomenon of birth. Moreover, practices and discourses of birth can be theorized in terms of the relationships between nature, body, and culture. How birthing is perceived and experienced strongly depends on social conditions, cultural norms, and gendered concepts, which determine whether it is an individual event or a collective matter. Thus, at the birth of every society and throughout its reproduction—in the truest sense of the word—there is birth.

This special issue seeks to refresh and expand the sociological perspectives on childbirth. The cultural-sociological description of childbearing as “techniques du corps” presented by Marcel Mauss is based on the assumption that birth is culturally variable and anything but “natural”, that it is rather the result of social preformation. Mauss describes the bodily techniques of childbearing as “the ways in which people in one society and in another traditionally use their bodies” (Mauss 2010 [1935], p. 219 f.). Moreover, if one takes seriously the cultural theoretical perspective that orders of knowledge enable and constrain social practices, it makes sense to devote attention to these knowledge orders (Reckwitz 2000). This is precisely what we want to do in this special issue “Cultures of Birthing in Transition”.

The COVID-19 pandemic has heavily disrupted societal spheres and exacerbated social inequity. These ruptures have impacted health care delivery, especially perinatal care protocols. Around the world, rapid changes have brought tight restrictions and significant uncertainties that have affected pregnant and birthing persons. While these developments have been met with relatively little protest, birthing conditions in general are increasingly subjected to critique by families as well as professionals; a new discomfort with birth appears to have emerged. Since 2013, the so-called Roses Revolution, inspired by a worldwide movement, has been held annually in Europe on November 25. On this day, mothers arrange to place roses in front of birthing units. Each rose symbolizes a woman who has experienced “physical or psychological violence” during childbirth (Ricoy 2013). Activists associated with the Roses Revolution want to raise awareness about “traumatic experiences and trauma suffered during childbirth” (Traum(a)Geburt e. v. 2022) and have organized themselves in various associations in several major cities, primarily via blogs and YouTube or other social media. They also refer to the WHO Declaration on the prevention and elimination of disrespect and abuse during facility-based childbirth (World Health Organization 2015). Women who have found themselves traumatized by interventions during childbirth have found validation from various experts in psychotherapy and psychiatry (Harms 2016). In the field of parent-child psychotherapy, services for addressing birth trauma are increasing (Deyringer 2016). Follow-up problems, such as breastfeeding difficulties, depression, and attachment problems with the infant, are linked to this experience of violence with reference to psychological and psychotherapeutic knowledge. Attachment theory has been a particular source of inspiration in this respect. This topic has also received increasing attention from the press in recent years (e.g., SRF 2018; DLF 2017, 2018; Wienerin 2018).

The publications from the ranks of those affected distinguish between psychological and physical violence. Psychological violence during childbirth is defined as undignified or disrespectful treatment of a woman giving birth, such as the exertion of pressure or the creation of fear to achieve the woman’s “participation” or leaving the woman alone. Physical violence during childbirth includes physical interventions (e.g., episiotomy, manual stretching of the cervix, cesarean section, vacuum extraction, or forceps delivery) that are unnecessary and not medically indicated, unnecessarily frequent or unnecessarily rough or painful examinations, gross extraction or tearing of the placenta, the so-called Kristeller maneuver (fundal pressure during active birth to allegedly shorten the last stage of labor), unnecessary induction of labor with labor-promoting agents, and generally restricting the freedom of movement of the woman in labor when she experiences such restriction as unnecessary (e.g. by strapping her to leg restraints or using a continuous cardiotocography) (Imlau 2017). One of the first medical publications on the topic distinguishes four types of violence: omission, verbal violence (yelling at, threatening, or insulting the woman), physical violence (including denial of pain medication), and sexual violence (d’Oliveira et al. 2002). The countries in focus are Peru, Brazil, Tanzania, South Africa, and Nigeria. In Latin American countries, such as Argentina and Venezuela, “obstetric violence” is now treated as a legal issue and is usually addressed as a form of structural violence (Galtung 1969; Farmer 2004; Morales et al. 2018), such as racial discrimination or denial of treatment based on social origin. In the United States, where obstetric violence is also regarded as a legal issue (Kukura 2018), organizations such as Black Birthing Justice highlight racialized treatment structures and disparities between Black and white women regarding the risk of maternal or infant death during childbirth (Villarosa 2018). While, in these countries, activists and their advocates (lawyers, midwives, and doctors) are still struggling for women’s rights, in most European countries, legally enshrined standards exist to protect women from such abuses. Activists, social media commentators, and journalistic contributions agree that violence is being done to women and that the topic has been neglected so far (Mundlos 2015). Fathers, as witnesses and mediated victims, have a marginal role in this debate (Rose 2017). From a historical perspective, the debate seems like a recapitulation of the birth center movement of the 1980s. Both debates discuss the supposedly private activity of birth as a political event and criticize encroachments on women’s autonomy during childbirth and, above all, the medicalization of the birth process. The birth center movement fought for new places of birth and raised awareness of the topic, resulting in the variety of possibilities for childbirth found in European countries today, especially in northern Europe. Almost 40 years after these advances, the topic of obstetric trauma and its negative consequences for mothers is now re-emerging as a topic for debate.

In German-speaking countries, where the discussion is currently prominent, the concept of trauma is particularly central. In parallel with the trauma discourse, “doulas”, psychosocial birth companions, are becoming established and are now increasingly offering their services to women in Switzerland, Austria, and Germany (Doula CH 2021; Doulas in Austria 2021; Doulas in Deutschland e. V. 2022): “Doulas qualifies through strength, intuition, warmth and security. Doulas travel with you to planet birth” (Doulas in Austria 2021 [translated by the authors]). Historically, doulas are by no means new, but they have received increased attention and are in greater demand since the advent of debate about potentially traumatic birth experiences. More and more training institutes are offering advanced training in postpartum trauma care. Furthermore, it has been claimed that 33% of all women experience a “traumatic birth”, which, in this context, includes the experience of an unplanned cesarean section (Rothenberg 2018).

Pursuant to the debates among activists and the media on the topic, reports on traumatic birth experiences have emerged in various European countries. In France, a comprehensive report was commissioned by the Haut Conseil à l’égalité entre les femmes et les hommes (Bousquet et al. 2018). Empirical data on the numbers of such experiences of violence in German-speaking countries are not available. A statement of the Federal Council issued on February 20, 2019 reads as follows:

1. The Federal Office of Public Health (FOPH), the Federal Statistical Office (FSO) and the Swiss Health Observatory (Obsan) do not have suitable data on this topic. 2. The Federal Council has no evaluable data on practice in the gynecological and obstetric context, since quality assurance in this area is the responsibility of the professional societies. How the issues raised in the report from France could be analyzed for Switzerland would need to be examined in more depth in collaboration with the professional societies. 3. The medical statistics of hospitals (MS) contain data on the number of episiotomies performed during deliveries in Switzerland. In 2017, 9906 perineal incisions were performed during natural births. The trend is downward: while in 2012 an episiotomy was performed in 25 percent of natural births, in 2017 the figure was 17 percent (Schweizer Bundesrat 2019).

A “small question” posed to the Federal Government of Germany on November 28, 2018 yielded the following data:

For the area of employed midwives in clinics, the Federal Government does not have any data. The German Insurance Association (GDV) has data from freelance midwives (clinical and out-of-hospital) on birth injuries. According to the GDV, data for the years 2002 to 2014 were analyzed and actuarially evaluated as part of a calculation project. According to this, it does not make sense to show individual years due to the small number of claims. These fluctuate between six and 31 for the period from 2004 to 2014. On average, about 20 birth claims are reported per year to the professional liability of freelance midwives. (Deutscher Bundestag 2018)

In Austria, so far, only the Birth Alliance, in cooperation with other activist groups such as Birth Rape, has collected data. An official statement from the government has not yet been requested. The data situation gives the impression that the existing cases and the activist and media discourse surrounding them are divergent. However, quantitative recording of the phenomenon is incomplete.

In accordance with our guiding assumption, all the developments described above point to a change in the self-perceptions of expectant parents—particularly mothers*—and an enormous discursive shift regarding the relevance of the birth experience for an individual’s own biography, which is changing birth cultures. But how is that so?

A summary of the social science contributions on the topic of birth and obstetric violence reveals seven dimensions that can give first approximations to a possible answer. First, we are dealing with the increasing medicalization and mechanization of obstetrics, which is reflected in the change in places of birth (more hospital births and fewer home births) and an increasing rate of intervention, especially cesarean sections, which may explain the increase in obstetric violence. This is related to the second dimension, which is the general economization of clinics. This includes diagnosis-related fixed-sum billing and the privatization of clinics, which also affect the staff and result in clinics being run according to an entrepreneurial logic (Jung 2018). Professionals are limited in their capacity to perform professionally due to time and efficiency pressures, which may lead to an increase in treatment errors or unprofessional actions. At the same time, the “claim for jurisdiction” (Abbott 1988) expressed by those involved plays a role in this (Jung 2018); different perspectives on birth and different liabilities can affect teamwork and can negatively impact women’s feelings of security while giving birth. In parallel with this economization of clinics, the employment conditions of midwives and obstetricians are changing due to stricter requirements for liability insurance. Third, in connection with the explanations just presented, ethnographic studies have shown that experiences of reification and bodily fragmentation are increasingly being reported by childbearing persons (Rose et al. 2017; Chadwick 2017), which confirms Duden’s (2002) thesis of disembodiment. Chadwick associates the production of docile bodies and control over resistant bodies through obstetric violence as the result of assembled gendered idealizations of the “good mother” or “good woman” and medical imperatives about “good patients”, which contrast with emerging “powerful forms of embodiment” (Chadwick 2017, p. 501). Fourth, these developments go hand in hand with a re-naturalization of the birthing process, which implies an emphasis on binary concepts of gender: “The natural intuition of the woman should be strengthened again” (Doulas in Deutschland e. V. 2022), and mother and child should stand again as a “unit” at the center of obstetrics. The emphasis on the “rediscovery” of “intuitive female knowledge” of the body can also be described as a spiritualization of birth. New phenomena, such as professionally unassisted births in water, the forest, or other places in “nature”, have emerged, which, while few in number, are widely documented on social media. This has resulted in the establishment of a discourse of “naturalness” that promotes undisturbed, intervention-free, and therefore safe childbirth without undesirable outcomes for the birthing person and their child. Nature is romanticized and perceived as something purely positive, while potential negative “natural” outcomes are disregarded. This may contribute to the expectations of pregnant persons and their companions, as well as clinics and birth centers, remaining unfulfilled. Fifth, the social science literature has described a shift in birth cultures toward claims of self-actualization and the perception of birth as a project (Seehaus 2015; Villa et al. 2011). The birth experience is presented as a “quasi-transcendent experience” (Rose and Schmied-Knittel 2011) and a biographical event. This enormous change in perceptions of the birth event may contribute to disappointment with the clinical experience, which is experienced as abusive, violent, or traumatic in relation to the idealized course of events. This development includes a paradoxical simultaneous increase in both the autonomy of pregnant and birthing persons (in terms of freedom of choice) and their self-responsibilization. Sixth, there are also signs of a general reactualization of feminist discourses around self-determination and gender. In particular, as part of the #MeToo debate and the media coverage of it, dismaying reports emerged about practices such as the “husband stitch”, which refers to tighter stitching of the perineum after an episiotomy, may have created a new feminist consciousness around the theme of “#MeToo in the delivery room” (Wienerin 2018). The seventh and final dimension concerns societal pluralization, which can result in cultural differences between expecting families and health professionals and structural barriers to the finding of common ground, especially in vulnerable moments such as birth (Kennedy et al. 2006), resulting in care relations being prone to misunderstanding.

In conclusion, notions of normality regarding birth experiences seem to be shifting. The painless, complication-free, and as natural as possible birth that some expect today is in contrast with the reality in delivery rooms, where structural resources, especially time for individualized care, are scarce. This can leave subjects feeling disappointed, alienated, or even traumatized. It seems that the professions involved in birth today are no longer only responsible for attending births (midwives/doulas) and the medical care of women during birth (physicians) but are also expected to be responsible for a good and successful birth experience (cf. Jung 2017). This special issue aims to investigate this change. However, the aim is not to discredit the articulations of affected subjects or to deny the reality of their suffering or the expertise of the relevant professions; rather, the question is how “traumatic birth” can establish itself in the Foucauldian sense as a dispositive in times that offer childbearing women more autonomy, security, and “natural” birthing possibilities than ever before. What “practices of translation” (Brunner 2014, p. 12) are undertaken here, and what is translated? What exactly is meant by “abuse” in childbirth, and what is considered a “good” birth? What assumptions of normality are associated with the birth experience? The articles included in this issue address these questions in various ways.

Lotte Rose’s contribution deals with the dispositives of responsibilization, optimization, and neoliberalization in the context of birth and thereby constituted (new and old) gender orders. Sarah Eckard empirically examines these dispositives, as well as others such as self-responsibility and risk, as subjects of individual birth experiences and interpretations. Christiane Winkler and Emine Babac address childbirth and birth experiences from an intersectional perspective, especially with regard to inequalities in health care for pregnant women and women giving birth and their implications for obstetric practice. Julia Böcker examines contemporary discourses and experiences related to stillbirth, and Mariette Mayrhofer-Deak reviews the monograph *Bodies that Birth* (2018) by sociologist Rachelle Chadwick and Sarah Eckardt reviews the edited volume on “feminist perspectives on parenthood” (2022) by Lisa Y. Haller and Alicia Schlender. We wish to continue our exchanges and collaborations on this subject and particularly look forward to future meetings in person, which had to be deferred due to the pandemic.
